# Risk factors for raised left ventricular filling pressure by cardiovascular magnetic resonance: Prognostic insights

**DOI:** 10.1002/ehf2.15011

**Published:** 2024-08-12

**Authors:** Ross J. Thomson, Ciaran Grafton‐Clarke, Gareth Matthews, Peter P. Swoboda, Andrew J. Swift, Alejandro Frangi, Steffen E. Petersen, Nay Aung, Pankaj Garg

**Affiliations:** ^1^ William Harvey Research Institute, NIHR Barts Biomedical Research Centre Queen Mary University of London London UK; ^2^ Barts Heart Centre, St Bartholomew's Hospital, Barts NHS Trust, West Smithfield London UK; ^3^ Norwich Medical School University of East Anglia Norwich UK; ^4^ Norfolk and Norwich University Hospitals Norwich UK; ^5^ The Institute of Cardiovascular and Metabolic Medicine University of Leeds Leeds UK; ^6^ Department of Infection, Immunity and Cardiovascular Disease The University of Sheffield Sheffield UK; ^7^ School of Computing University of Leeds Leeds UK; ^8^ Health Data Research UK London UK; ^9^ Alan Turing Institute London UK

**Keywords:** cardiovascular magnetic resonance, MRI, left ventricular filling pressure, heart failure epidemiology

## Abstract

**Background:**

Cardiovascular magnetic resonance (CMR) imaging shows promise in estimating pulmonary capillary wedge pressure (PCWP) non‐invasively. At the population level, the prognostic role of CMR‐modelled PCWP remains unknown. Furthermore, the relationship between CMR‐modelled PCWP and established risk factors for cardiovascular disease has not been well characterized.

**Objective:**

The main aim of this study was to investigate the prognostic value of CMR‐modelled PCWP at the population level.

**Methods:**

Employing data from the imaging substudy of the UK Biobank, a very large prospective population‐based cohort study, CMR‐modelled PCWP was calculated using a model incorporating left atrial volume, left ventricular mass and sex. Logistic regression explored the relationships between typical cardiovascular risk factors and raised CMR‐modelled PCWP (≥15 mmHg). Cox regression was used to examine the impact of typical risk factors and CMR‐modelled PCWP on heart failure (HF) and major adverse cardiovascular events (MACE).

**Results:**

Data from 39 163 participants were included in the study. Median age of all participants was 64 years (inter‐quartile range: 58 to 70), and 47% were males. Clinical characteristics independently associated with raised CMR‐modelled PCWP included hypertension [odds ratio (OR) 1.57, 95% confidence interval (CI) 1.44–1.70, *P* < 0.001], body mass index (BMI) [OR 1.57, 95% CI 1.52–1.62, per standard deviation (*SD*) increment, *P* < 0.001], male sex (OR 1.37, 95% CI 1.26–1.47, *P* < 0.001), age (OR 1.33, 95% CI 1.27–1.41, per decade increment, *P* < 0.001) and regular alcohol consumption (OR 1.10, 95% CI 1.02–1.19, *P* = 0.012). After adjusting for potential confounders, CMR‐modelled PCWP was independently associated with incident HF [hazard ratio (HR) 2.91, 95% CI 2.07–4.07, *P* < 0.001] and MACE (HR 1.48, 95% CI 1.16–1.89, *P* = 0.002).

**Conclusions:**

Raised CMR‐modelled PCWP is an independent risk factor for incident HF and MACE. CMR‐modelled PCWP should be incorporated into routine CMR reports to guide HF diagnosis and further management.

## Graphical abstract

In this population‐based study we demonstrate the prognostic relevance of CMR‐modelled PCWP to predict incident HF and MACE and also risk factors associated with it. Cardiovascular magnetic resonance imaging (CMR) shows promise in estimating pulmonary capillary wedge pressure (PCWP) non‐invasively. This study investigated the prognostic role of CMR‐modelled PCWP at the population level in 39 163 participants from the UK Biobank. CMR‐modelled PCWP was calculated using a model incorporating left atrial volume, left ventricular mass and sex. Risk factors associated with elevated CMR‐modelled PCWP were: hypertension (OR 1.57, *P* < 0.001), BMI (OR 1.57, per *SD* increment, *P* < 0.001), male sex (OR 1.37, 95%, *P* < 0.001), age (OR 1.33, per decade increment, *P* < 0.001) and regular alcohol consumption (OR 1.10, *P* = 0.012). CMR‐modelled PCWP was independently associated with the incidence of HF (HR 2.91, *P* < 0.001) and MACE (HR 1.48, *P* = 0.002).

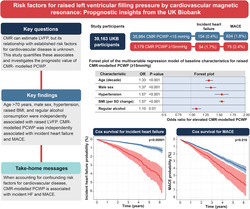

## Introduction

The prevalence of heart failure (HF) is increasing, primarily due to an ageing population and better treatment of cardiovascular conditions (e.g., ischaemic heart disease) that can give rise to HF.^1^ Despite advances in managing cardiovascular disease, the prognosis of patients with HF remains poor, with quality of life markedly reduced.^2^ Following the initial diagnosis, HF patients are hospitalized once per year on average, and two in three will not be alive 5 years following diagnosis.^3^, ^4^ The hallmark of HF is raised left ventricular (LV) filling pressure (LVFP). This can be estimated from pulmonary capillary wedge pressure (PCWP), which can be measured directly using the reference standard, cardiac catheterization. Invasive assessment is not feasible or required for most patients with suspected HF, and LVFP is conventionally evaluated using multiparametric echocardiography.^5^

With its high spatial resolution, excellent signal‐to‐noise ratio and inherent tomographic nature, cardiovascular magnetic resonance (CMR) imaging is evolving into a valuable tool in the diagnostic workflow of patients with suspected HF.^6^ CMR is the gold‐standard imaging technique for quantifying ventricular volumes and myocardial mass, and for assessing tissue characterization, and regional and global systolic function. Several LV diastolic function indices can be derived from CMR, analogous to echocardiography, including myocardial deformation using strain analysis and phase‐contrast CMR‐derived transmitral and pulmonary venous flow.^7^ These indices require sophisticated postprocessing and frequently dedicated imaging sequences. LV mass and left atrial volume (LAV) are parameters readily obtainable by CMR that have been shown to correlate independently with invasively measured PCWP in patients with breathlessness.^8^

We have previously published a CMR‐derived model predicting PCWP using LV mass and maximum LAV, which has exhibited good specificity (92%) and negative predictive value (78%) for dichotomous identification of raised invasive PCWP (≥15 mmHg).^8^ Of value clinically, CMR‐modelled PCWP demonstrated superior concordance to invasive assessment in classifying patients as ‘normal’ or ‘raised’ PCWP compared with echocardiography (76% vs. 25%). Further, there is the suggestion that CMR‐modelled PCWP may be superior to invasive measurement to predict mortality. This may relate to the chronic influence of LVFP on left atrial size rather than snapshots captured during cardiac catheterization.^8^, ^9^ There is a need to validate CMR‐modelled PCWP in external cohorts, understand the factors influencing LVFP and evaluate its utility as a cardiac biomarker in predicting clinically important outcomes in larger cohorts.

This prospective population‐based cohort study used data from the UK Biobank (UKB) and had two primary aims: first, to evaluate and quantify the relationship between established risk factors for cardiovascular disease and CMR‐modelled PCWP; second, to investigate the prognostic value of raised CMR‐modelled PCWP.

## Methods

### UK Biobank

UKB is a health research resource of major international importance.^10^ It is a prospective cohort study with deep genetic, lifestyle, physical, and health data collected on ~500 000 people aged 40–69 years at enrolment.^11^ Initial enrolment lasted 4 years from 2006, and participants will be followed‐up for at least 30 years. The data from laboratory tests, imaging investigations, and genetic analysis are accessible to researchers undertaking health‐related research in the public interest.

Linkages with hospital episode statistics and death registers allow prospective tracking of health outcomes for all participants, documented according to the International Classification of Disease codes (*Table*s S1 and S2). UKB has also produced algorithmically defined outcomes for key illnesses, such as acute myocardial infarction, which integrate data from several sources.^12^

### Study population

The 43 666 participants who entered the imaging substudy of the UKB between April 2014 and March 2020 were included in this study. The North West Multi‐Centre Research Ethics Committee approved the UKB study in June 2011 (11/NW/0382), which was extended on 18 June 2021 (21/NW/0157). All individuals provided informed consent at the time of enrolment in UKB.

### Cardiovascular magnetic resonance imaging

The UKB CMR imaging protocol and image analysis have been described previously.^13^, ^14^ All participants underwent CMR using a 1.5 Tesla scanner (MAGNETOM Aera, Syngo Platform VD13A, Siemens Healthcare, Erlangen, Germany). For cardiac function, three long‐axis cines (two‐, three‐ and four‐chamber) and a complete short‐axis stack covering the left ventricle (LV) and right ventricle (RV) were acquired at one slice per breath‐hold. All acquisitions used balanced steady‐state free precession with typical parameters: TR/TE = 2.6/1.1 ms, flip angle 80°, Grappa factor 2, voxel size 1.8 × 1.8 × 8 mm (6 mm for long‐axis). The actual temporal resolution of 32 ms was interpolated to 50 phases per cardiac cycle (~20 ms).

### CMR image analysis

As described in detail elsewhere, a standard operating procedure for the analysis of each cardiac chamber was developed.^14^ RV endocardial borders, LV endocardial and epicardial borders, and LA endocardial borders were manually traced in the end‐diastolic and end‐systolic phases for the first 5065 participants. This allowed the creation of ground‐truth contours for atrial and ventricular volumes and LV mass. LV papillary muscles were included as part of LV end‐diastolic and end‐systolic volumes and excluded from LV mass (LVM) to reduce observer variability. LAV was calculated using the biplane area‐length method. A deep learning algorithm was trained to derive LV volumetric parameters and LVM using these manually annotated images. Using a three‐dimensional statistical shape model embedded in a deep neural network, the LA was automatically segmented in both four‐ and two‐chamber views. These methods and quality control processes have been reported previously, where segmentation performance accuracy is comparable to human experts.^15^ These algorithms were deployed at scale for the remainder of the participants within the UKB imaging sub study to derive LAV and LVM.^16^ A model to estimate PCWP using sex and CMR‐obtained LVM and LAV was applied to the UKB cohort (Supporting information Methods).^17^

### Statistical analysis

Continuous variables are reported as medians with inter‐quartile ranges (IQR). Categorical variables are reported as numbers and percentages. The baseline characteristics of the included participants were stratified by CMR‐modelled PCWP and compared using one‐way ANOVA statistics.

Statistical analysis was performed using R (version 4.1.1) in RStudio Server (version 2022.12.0). Restricted cubic spline regression was performed using the RMS package, and survival analysis was performed using the ‘survminer’ and adjusted Curves packages.

### Regression analyses

Treating CMR‐modelled PCWP as a dichotomous variable (<15 vs. ≥15 mmHg), univariable logistic regression was used to explore the relationships between PCWP and typical risk factors for cardiovascular disease. Variables significantly associated with increased odds of elevated PCWP in univariable logistic regression models were taken forward into a multivariable model.

Because preliminary analysis indicated that the relationship between PCWP and age was non‐linear, models using ordinal least squares regression with restricted cubic splines were constructed. This allowed the relationship between CMR‐modelled PCWP and age to be modelled semi‐independently across different age segments (knots). The data were partitioned into a training set (75%) and a validation (25%) set. The optimal number of knots was determined through iteration on the training set to minimize the Akaike information criterion (AIC). The AIC measures relative model performance, balancing the goodness of fit against model complexity. The performance of models using the optimal number of knots was assessed in both the training and validation sets. Residual mean square error, mean absolute error and *R* ^2^ statistic were used to compare model performance between the two datasets.

Multivariable ordinary least squares regression was used to model the relationship between CMR‐modelled PCWP and age (restricted cubic spline with nine knots) in the training set, adjusted for typical cardiovascular risk factors. The modelled relationship was visually inspected to identify an inflection point, defined as the abrupt change in the relationship between CMR‐modelled PCWP and age. The modelling process was repeated in the validation set to confirm the shape of this relationship and the position of the inflection point. The age at this inflection point was used as the dichotomized age variable for subsequent analyses.

To understand the risk of elevated PCWP in individuals with different combinations of risk factors, a multivariable logistic regression model was constructed to predict the probability of elevated PCWP given age (modelled using a restricted cubic spline with nine knots), systolic blood pressure (SBP), sex and the presence of obesity. The absolute risk of elevated PCWP was calculated for males and females, obese and non‐obese, for each decade of age (from 40 to 80 years) and each 20 mmHg increment of SBP (from 120 to 180 mmHg). The relative risk for each increment of age and SBP, in the presence and absence of obesity, was calculated separately for males and females compared with the risk of the group with the lowest SBP and age. The results were presented within a nomogram.

### Survival analysis

Univariable Cox proportional hazards regression was used to explore the impact of typical risk factors for cardiovascular disease, and CMR‐modelled PCWP, on the primary outcomes of incident HF and major adverse cardiovascular events (MACE). After adjusting for potential confounders, multivariable Cox regression models were created to explore the impact of CMR‐modelled PCWP on HF and MACE. The assumption of proportional hazards was assessed by visually examining the scaled Schoenfeld residuals.

The significance threshold was set at *P* < 0.05.

### Outcomes

This study investigated two primary outcomes: incident HF and MACE. Incident HF was defined as patients who presented with HF symptoms and signs to emergency departments and needed hospital admission for decompensated HF. MACE was defined as the composite outcome of the following recorded outcomes: non‐fatal myocardial infarction, non‐fatal stroke and cardiovascular death.

## Results

### Study population

CMR imaging data were available for 43 666 participants. There were 4503 participants excluded because the image quality precluded the determination of LVM or LAV. Then 39 163 participants were included in the study. The baseline characteristics are summarized in *Table* 1, stratified by CMR‐modelled PCWP. Of these, 3179 (8.1%) participants had raised CMR‐modelled PCWP. The median age of all participants was 64 years (IQR 58 to 70), and 18 465 (47%) were male.

**Table 1:** Baseline characteristics stratified by cardiovascular magnetic resonance‐modelled left ventricular filling pressure.

Characteristic	All participants	LVFP < 15 mmHg	LVFP ≥ 15 mmHg	*P* value
	*n* = 39 163	*n* = 35 984	*n* = 3179	
Age (years)^a^	64 (58, 70)	64 (58, 70)	66 (59, 72)	<0.001
Male sex, *n* (%)	18 465 (47)	16 641 (46)	1824 (57)	<0.001
White ethnicity, *n* (%)	37 856 (97)	34 782 (97)	3074 (97)	>0.9
Body mass index (kg/m^2^)^a^	25.2 (22.9, 28.1)	25.1 (22.8, 27.8)	27.1 (24.4, 30.5)	<0.001
Smoking				
Never, *n* (%)	24 301 (63)	22 435 (63)	1866 (59)	<0.001^b^
Previous, *n* (%)	13 172 (34)	12 007 (34)	1165 (37)	
Current, *n* (%)	1309 (3.4)	1194 (3.4)	115 (3.7)	
Alcohol				
Regular alcohol intake^c^, *n* (%)	17 633 (45)	16 135 (45)	1498 (47)	0.014
Comorbidities				
Diabetes mellitus, *n* (%)	2213 (5.7)	1957 (5.4)	256 (8.1)	<0.001
Hypertension, *n* (%)	12 362 (32)	10 840 (30)	1522 (48)	<0.001
Hyperlipidaemia, *n* (%)	13 633 (35)	12 344 (34)	1289 (41)	<0.001

Abbreviation: LVFP, left ventricular filling pressure;

^a^ Median (inter‐quartile range).

^b^ One‐way ANOVA.

^c^ At least three times a week.

Participants with raised CMR‐modelled PCWP were older than participants with normal CMR‐modelled PCWP (66 years, IQR 59 to 72, vs. 64 years, IQR 58 to 70, *P* < 0.001), more frequently male (57% vs. 46%, *P* < 0.001), reported a higher frequency of smoking history (40% vs. 37%, *P* < 0.001), were more frequently regular consumers of alcohol (47% vs. 45%, *P* = 0.01) and had a higher body mass index (BMI) (27.1 kg/m^2^, IQR 24.4 to 30.5, vs. 25.1 kg/m^2^, IQR 22.8–27.8). Those with raised CMR‐modelled PCWP, in comparison with participants with normal CMR‐modelled PCWP, were found to have a higher frequency of diabetes (8.1% vs. 5.4%, *P* < 0.001), hypertension (48% vs. 30%, *P* < 0.001), and hyperlipidaemia (41% vs. 34%, *P* < 0.001).

### Factors influencing CMR‐modelled PCWP

The primary continuous variables that exhibited a significant correlation with CMR PCWP included the following: SBP [*β* = 0.02, *R* = 0.23, regression equation: 9.9 + 0.02*x*, 95% confidence interval (CI) 0.019, 0.021, *P* < 0.001], diastolic blood pressure (*β* = 0.015, *R* = 0.1, regression equation: 12 + 0.015*x*, 95% CI 0.013, 0.017, *P* < 0.001) and BMI (*β* = 0.098, *R* = 0.25, regression equation: 10 + 0.098*x*, 95% CI 0.094, 0.102, *P* < 0.001).

Treating CMR‐modelled PCWP as a dichotomous outcome variable (<15 mmHg vs. ≥15 mmHg) in univariable logistic regression models, all exposure variables analysed, excluding ethnicity, were associated with CMR‐modelled PCWP (*Table* 2). In descending order by odds ratio (OR), these included hypertension (OR 2.13, 95% CI 1.98–2.29, *P* < 0.001), BMI (OR 1.58, 95% CI 1.53–1.63, per standard deviation (*SD*) increment, P < 0.001), male sex (OR 1.56, 95% CI 1.45–1.68, P < 0.001), diabetes (OR 1.52, 95% CI 1.33–1.74, P < 0.001), age (OR 1.33, 95% CI 1.27–1.40, per decade increment, P < 0.001), hyperlipidaemia (OR 1.31, 95% CI 1.21–1.41, *P* < 0.001), positive smoking history (OR 1.13, 95% CI 1.05–1.21, *P* = 0.002), and regular alcohol consumption (OR 1.10, 95% CI 1.02–1.18, P = 0.01).

**Table 2:** Univariable logistic regression model of risk factors for raised cardiovascular magnetic resonance‐modelling pulmonary capillary wedge pressure (≥15 mmHg).

Characteristic	*n*	OR	95% CI	*P* value
Age (per decade increment)	39 163	1.33	1.27, 1.40	<0.001
Male sex	39 163	1.56	1.45, 1.68	<0.001
White ethnicity	39 152	1.00	0.82, 1.23	>0.9
Body mass index per *SD* increment	39 163	1.58	1.53, 1.63	<0.001
Previous or current smoker	39 078	1.13	1.05, 1.21	0.002
Diabetes	39,163	1.52	1.33, 1.74	<0.001
Hypertension	39 163	2.13	1.98, 2.29	<0.001
Hyperlipidaemia	39 163	1.31	1.21, 1.41	<0.001
Regular alcohol consumption	39 163	1.10	1.02, 1.18	0.013

Abbreviations: CI, confidence interval; OR, odds ratio; *SD*, standard deviation.

**Figure 1:**
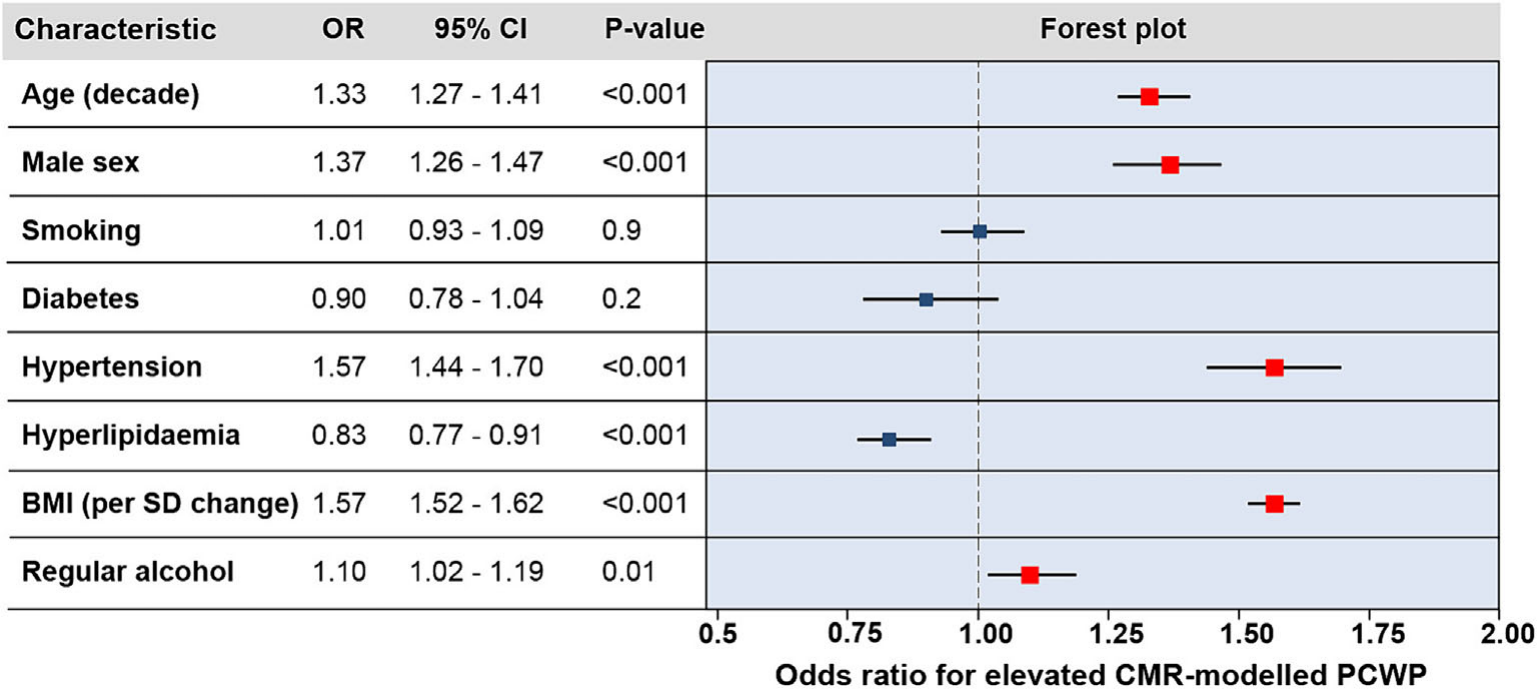
Forest plot showing the relationships between baseline characteristics and raised CMR‐modelled PCWP, as determined by multivariable logistic regression (≥15 mmHg). BMI, body mass index; CI, confidence interval; CMR, cardiovascular magnetic resonance; OR, odds ratio; PCWP, pulmonary capillary wedge pressure; *SD*, standard deviation.

In multivariable logistic regression modelling, five factors were positively associated with elevated CMR‐modelled PCWP: hypertension (OR 1.57, 95% CI 1.44–1.70, *P* < 0.001), BMI (OR 1.57, 95% CI 1.52–1.62, per *SD* increment, *P* < 0.001), male sex (OR 1.37, 95% CI 1.26–1.47, *P* < 0.001), age (OR 1.33, 95% CI 1.27–1.41, per decade increment, *P* < 0.001), and regular alcohol consumption (OR 1.10, 95% CI 1.02–1.19, *P* = 0.012) (*Figure* 1).

### Age and CMR‐modelled PCWP

**Figure 2:**
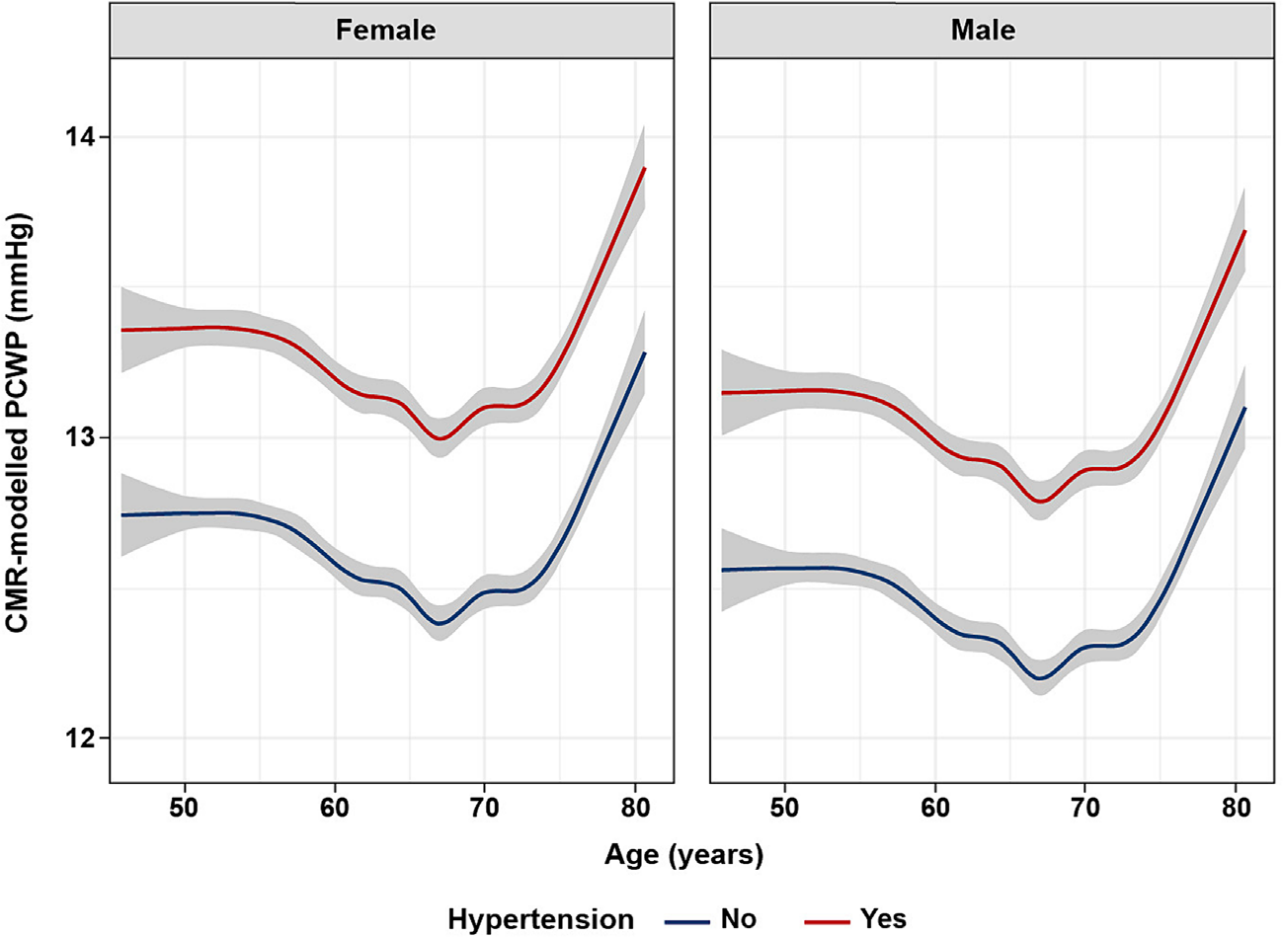
Relationships between CMR‐modelled PCWP and age modelled using a cubic spline of age with nine knots, adjusted for all covariables. Hypertension shifts the curves similarly in both female and male UK Biobank participants, and the age dependency with an inflection point at age 70 is similar in both sexes. CMR, cardiovascular magnetic resonance; PCWP, pulmonary capillary wedge pressure.

When the relationship between CMR‐modelled PCWP and age was modelled using a restricted cubic spline of age with nine knots, adjusted for all covariables studied, there was little correlation between CMR‐modelled PCWP and age until around 70 years, after which CMR‐modelled PCWP increased rapidly with age (*Figure* 2). The shape of this relationship was confirmed through repeat modelling in the holdout validation set (Table S3).

### Effect of CMR‐modelled PCWP and typical cardiovascular risk factors on incident HF and MACE

In univariable Cox regression models, four of the factors associated with raised CMR‐modelled PCWP in multivariable logistic regression modelling, and raised CMR‐modelled PCWP itself, were associated with an increased hazard for incident HF: elevated CMR‐modelled PCWP (HR 5.76, 95% CI 4.22–7.88, *P* < 0.001), hypertension (HR 4.09, 95% CI 3.08–5.43, *P* < 0.001), age (HR 3.35, 95% CI 2.70–4.16, per decade increment, *P* < 0.001), male sex (HR 2.04, 95% CI 1.54–2.70, *P* < 0.001) and BMI (HR 1.35, 95% CI 1.21–1.51, per *SD* increment, *P* < 0.001) (*Table* 3). Higher LV ejection fraction was associated with lower hazard of incident HF (HR 0.88, 95% CI 0.87–0.90, *P* < 0.001). Similar results were obtained for incident MACE (*Table* 3).

**Table 3:** Univariable logistic regression model for factors associated with an increased hazard for incident heart failure and MACE.

Characteristic	*n*	HR	95% CI	*P* value
Outcome: Incident heart failure				
Age (per decade)	38 958	3.35	2.70, 4.16	<0.001
Male sex	38 958	2.04	1.54, 2.70	<0.001
Hypertension	38 958	4.09	3.08, 5.43	<0.001
Body mass index per *SD* increment	38 958	1.35	1.21, 1.51	<0.001
Regular alcohol consumption	38 958	0.83	0.63, 1.10	0.2
Elevated CMR‐modelled PCWP (≥15 mmHg)	38 958	5.76	4.22, 7.88	<0.001
LA volume (ml)	38 958	1.03	1.02, 1.03	<0.001
LV mass (g)	38 958	1.03	1.02, 1.03	<0.001
LV end‐diastolic volume (ml)	38 958	1.02	1.01, 1.02	<0.001
LV end‐systolic volume (ml)	38 958	1.03	1.03, 1.03	<0.001
LV ejection fraction (%)	38 958	0.88	0.87, 0.90	<0.001
Outcome: MACE				
Age (per decade)	37 200	2.19	1.97, 2.43	<0.001
Male sex	37 200	2.47	2.11, 2.89	<0.001
Hypertension	37 200	2.53	2.18, 2.93	<0.001
Body mass index per *SD* increment	37 200	1.19	1.11, 1.27	<0.001
Regular alcohol consumption	37 200	0.92	0.80, 1.07	0.3
Elevated CMR‐modelled PCWP (≥15 mmHg)	37 200	2.06	1.62, 2.61	<0.001
LA volume (mL)	37 200	1.01	1.00, 1.01	<0.001
LV mass (g)	37 200	1.02	1.02, 1.02	<0.001
LV end‐diastolic volume (mL)	37 200	1.01	1.00, 1.01	<0.001
LV end‐systolic volume (mL)	37 200	1.01	1.01, 1.02	<0.001
LV ejection fraction (%)	37 200	0.96	0.95, 0.98	<0.001

Abbreviations: CI, confidence interval; CMR, cardiovascular magnetic resonance; HR, hazard ratio; LA, left atrial; LV, left ventricular; MACE, major adverse cardiovascular event; PCWP, pulmonary capillary wedge pressure; *SD*, standard deviation.

**Figure 3:**
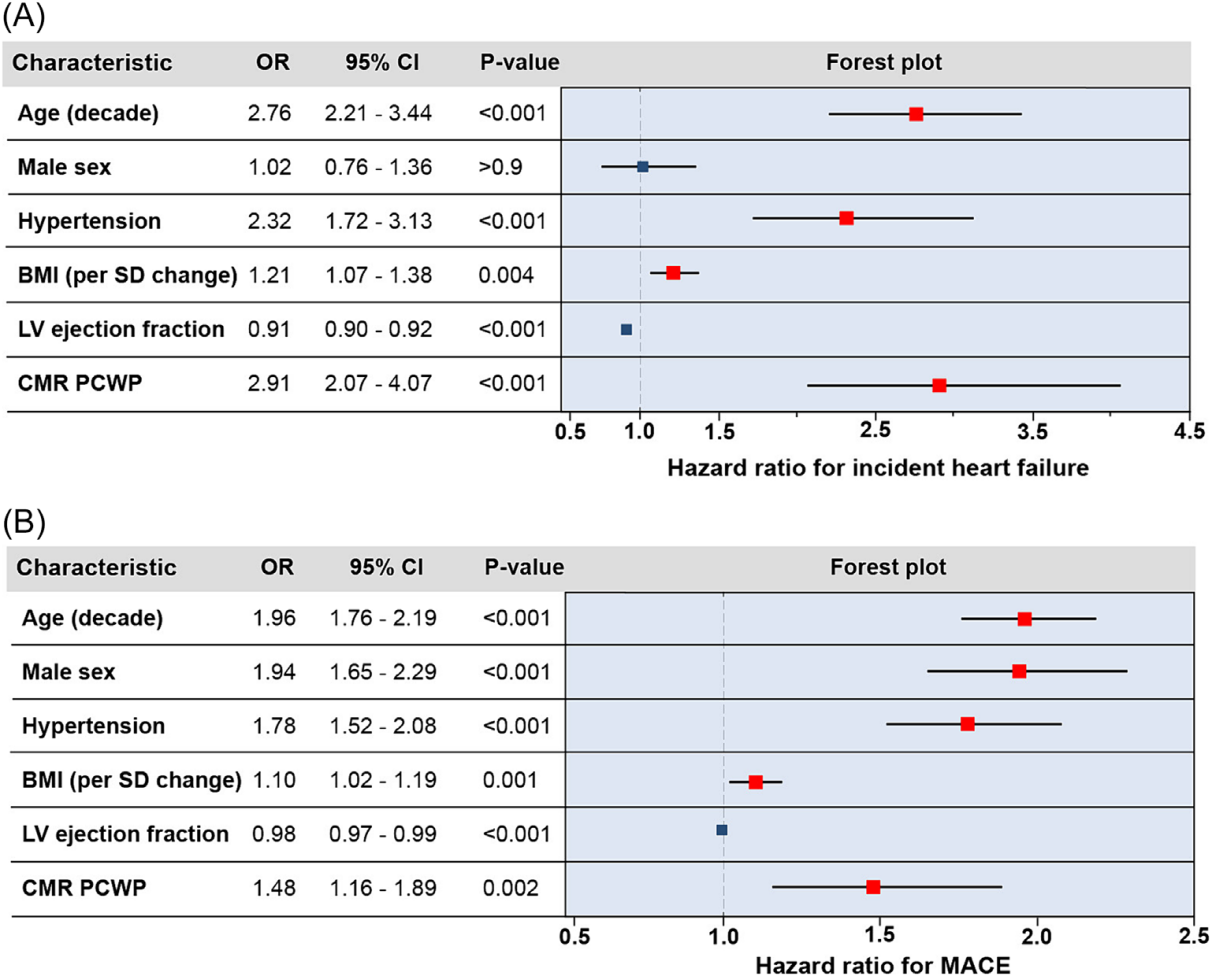
Forest plot showing the adjusted hazard ratios for incident outcomes across a range of exposures, as determined by multivariable Cox regression modelling: (A) HF; (B) MACE. BMI, body mass index; CI, confidence interval; CMR, cardiovascular magnetic resonance; LV, left ventricular; MACE, major adverse cardiovascular event; OR, odds ratio; PCWP, pulmonary capillary wedge pressure.

In a multivariable Cox regression model examining factors associated with incident HF (*Figure* 3A), age (HR 2.76, 95% CI 2.21–3.44, per decade increment, *P* < 0.001), hypertension (HR 2.32, 95% CI 1.72–3.13, *P* < 0.001), BMI (HR 1.21, 95% CI 1.07–1.38, per *SD* increment, *P* < 0.001), and elevated CMR‐modelled PCWP (HR 2.91, 95% CI 2.07–4.07, *P* < 0.001) were significantly associated with an increased hazard of incident HF. Higher LV ejection fraction was associated with a lower hazard of incident HF (HR 0.91, 95% CI 0.90–0.92, *P* < 0.001). Male sex was no longer significantly associated with incident HF.

In a multivariable Cox regression model examining factors associated with incident MACE (*Figure* 3B), age (HR 1.96, 95% CI 1.76–2.19, *P* < 0.001), male sex (HR 1.94, 95% CI 1.65–2.29), hypertension (HR 1.78, 95% CI 1.52–2.08), BMI (HR 1.10, 95% CI 1.02–1.19, per *SD* increment, *P* = 0.01) and elevated CMR‐modelled PCWP (HR 1.48, 95% CI 1.16–1.89, *P* = 0.002) were significantly associated with an increased hazard of MACE. Higher LV ejection fraction was associated with a lower hazard of incident MACE (HR 0.98, 95% CI 0.97–0.99, *P* < 0.001).

**Figure 4:**
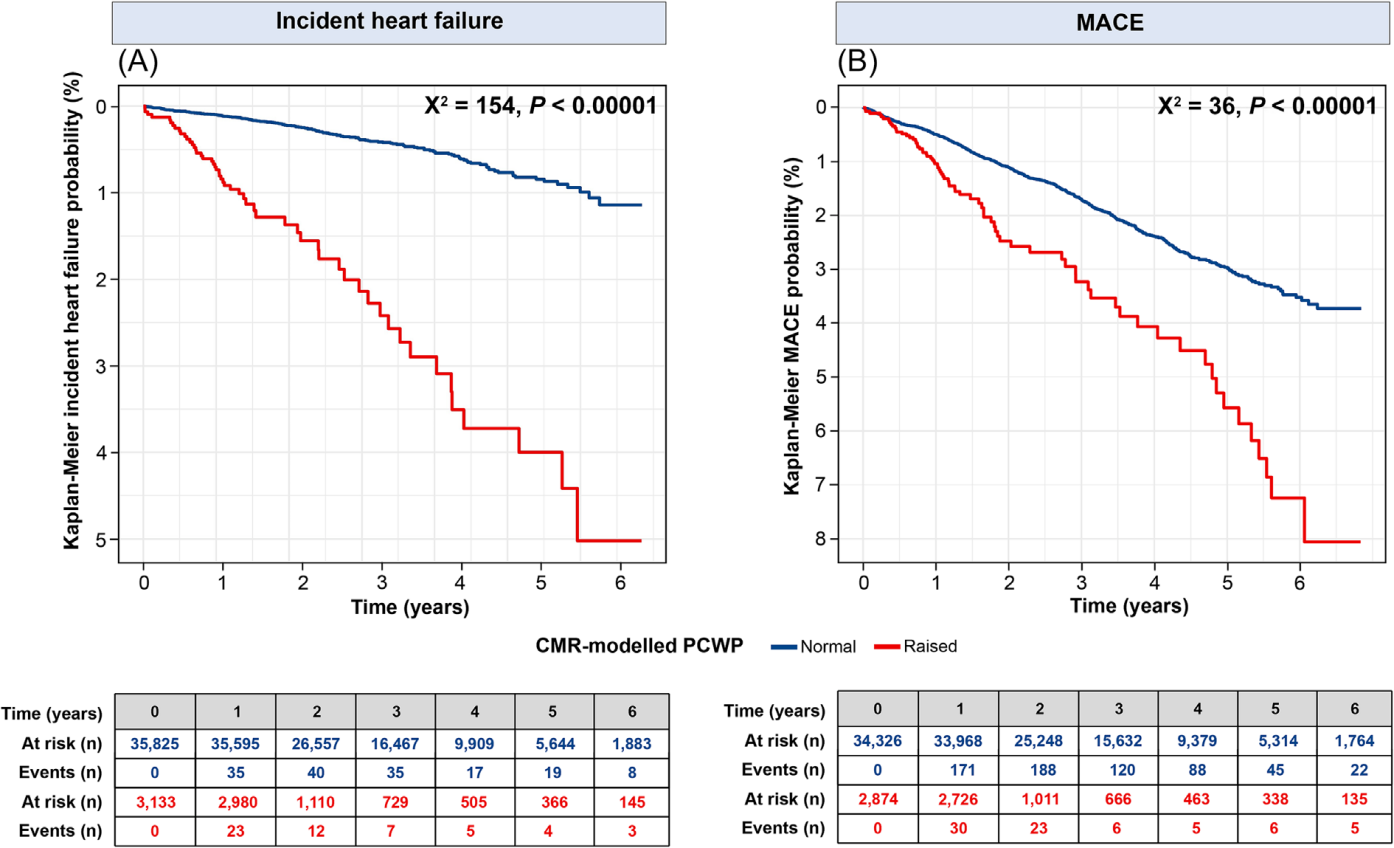
Kaplan–Meier survival curves stratified by CMR‐modelled PCWP. (A) Incident heart failure; (B) MACE. CMR, cardiovascular magnetic resonance; MACE, major adverse cardiovascular event; PCWP, pulmonary capillary wedge pressure.

**Figure 5:**
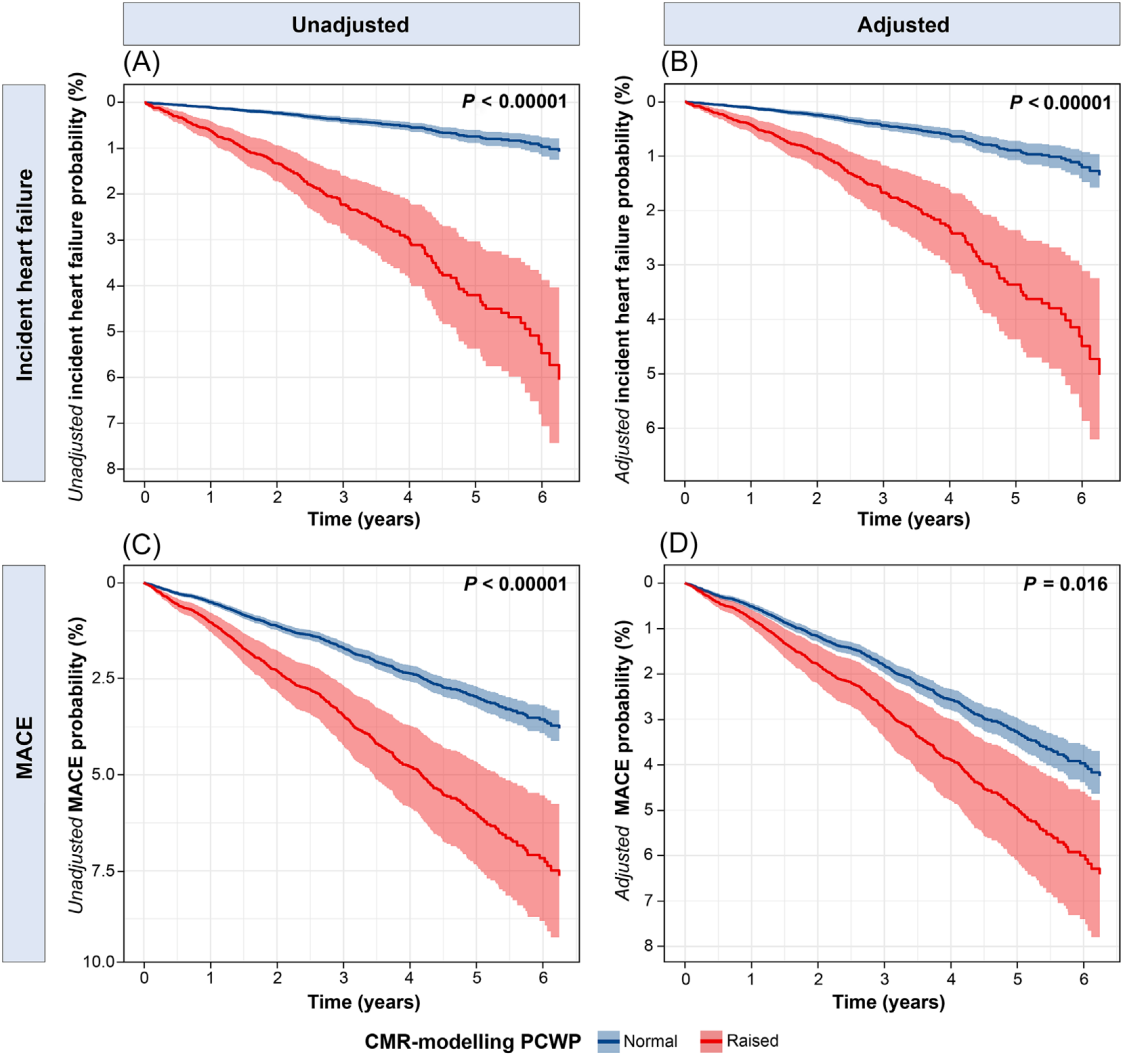
Cox survival curves (line, 95% confidence interval: shaded) stratified by the presence or absence of elevated CMR‐modelled PCWP. The left panel shows unadjusted survival for the two groups, from univariable regression Cox models, while the right panel shows survival adjusted for confounding variables using multivariable Cox models. (A) Incident heart failure; (B) adjusted incident heart failure; (C) MACE; (D) adjusted MACE. CMR, cardiovascular magnetic resonance; MACE, major adverse cardiovascular event; PCWP, pulmonary capillary wedge pressure.

In Kaplan–Meier survival analysis (*Figure* 4), individuals with raised CMR‐modelled PCWP were more likely to develop incident HF than those with normal CMR‐modelled PCWP at 6 year follow‐up (5% vs. 1%, *χ* ^2^ = 154, *P* < 0.00001). In addition, the probability of incident MACE was higher in individuals with elevated CMR‐modelled PCWP versus normal CMR‐modelled PCWP at 6 year follow‐up (8% vs. 4%, *χ* ^2^ = 36, *P* < 0.00001). Even after adjustment for confounding variables in the Cox‐regression curve analysis, individuals with elevated CMR‐modelled PCWP remained more likely to develop incident HF and experience MACE than those with normal CMR‐modelled PCWP. Cox curves for incident HF and MACE, stratified by the presence or absence of elevated CMR‐modelled PCWP, are presented in *Figure* 5.

**Figure 6:**
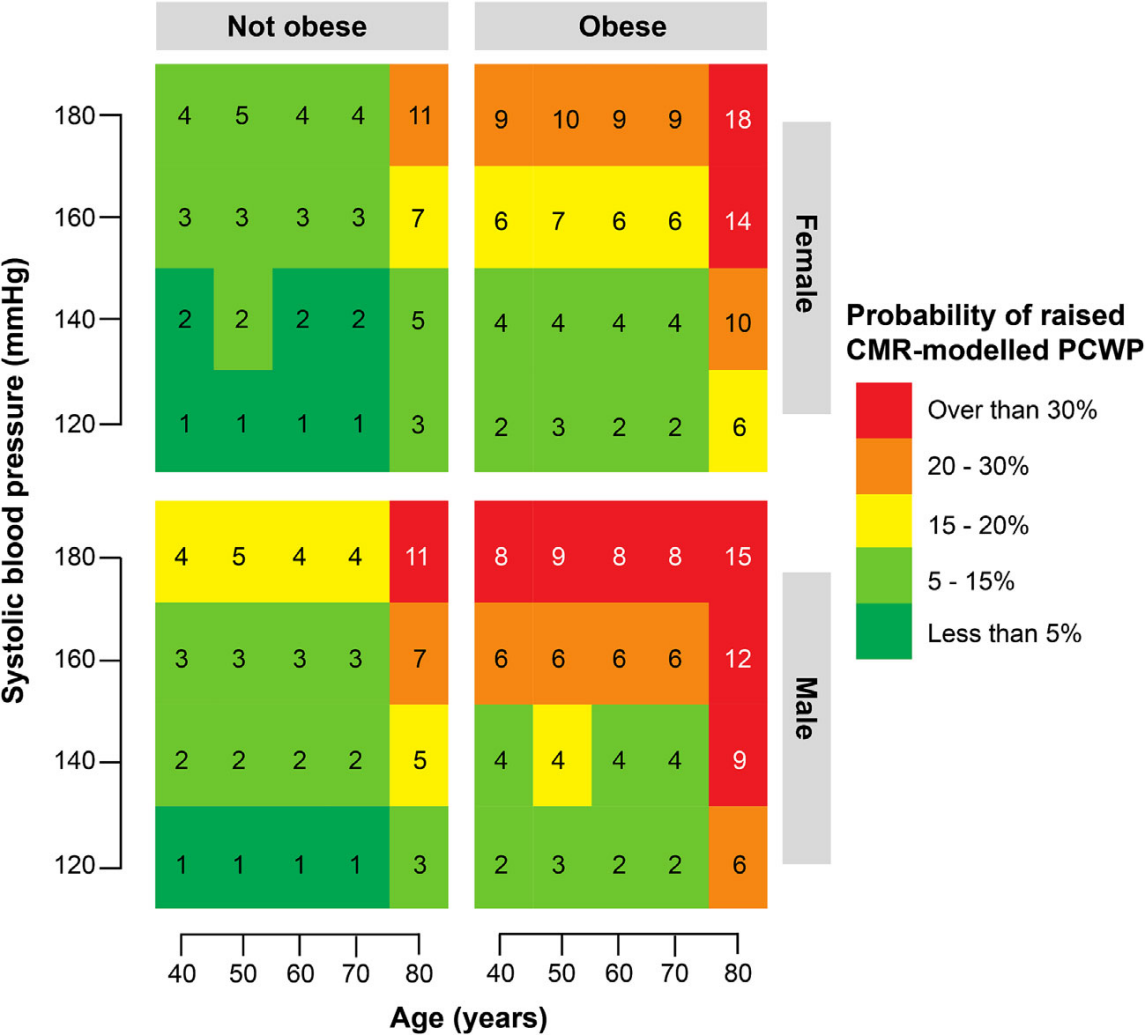
Risk prediction chart (nomogram) for raised left ventricular filling pressure. The nomogram shows the risk of elevated CMR‐modelled PCWP for different combinations of systolic blood pressure, age, sex, and presence/absence of obesity. The colour scale represents absolute risk, from low (green) to high (red). The number in each box is the relative risk for an individual with that combination of risk factors, compared with the lowest‐risk individual of their sex. CMR, cardiovascular magnetic resonance; PCWP, pulmonary capillary wedge pressure.

*Figure* 6 charts a risk‐prediction tool for raised CMR‐modelled PCWP that includes different grades of SBP, history of obesity, sex and age.

## Discussion

This large‐scale prospective cohort study of middle‐to‐older aged individuals identified multiple clinically important insights. First, age, male sex, hypertension, BMI and regular alcohol consumption were independently associated with higher CMR‐modelled PCWP. Second, a rapid rise in CMR‐modelled PCWP was observed above 70 years. Third, after accounting for confounding from other established cardiovascular risk factors, including LV ejection fraction, CMR‐modelled PCWP was found to be an independent predictor for incident HF and MACE. These findings may have implications for targeted population screening strategies and mitigation and public health measures to reduce the risk of HF development.

Elevated LVFP at rest or during exercise is the pathophysiological hallmark of HF. Aside from its diagnostic role, it has a specific value in identifying patients at increased risk of death, and targeted reduction of LVFP has been shown to reduce HF hospitalization.^18^, ^19^ Echocardiography is a first‐line imaging tool for the assessment of individuals presenting with breathlessness or other signs or symptoms suggestive of HF and allows for the detailed evaluation of cardiac function and morphology. Because individual echocardiographic parameters are poorly correlated with LV filling pressures, multiparametric diagnostic algorithms have been developed that integrate sophisticated functional and morphological data from echocardiography to evaluate diastolic function.^5^ Nonetheless, the diagnostic utility of integrated echocardiographic assessment has been debated, and the algorithm recommended by international guidelines has been found to perform poorly.^20^ There is, therefore, an unmet clinical need to identify complementary non‐invasive imaging methods that can risk‐stratify patients at higher risk for raised filling pressures and determine the prognostic significance of these stratifications. Our group has previously derived and validated a model to reliably estimate PCWP from CMR; this study expands on our previous work by demonstrating the prognostic value of CMR‐derived PCWP in a non‐selected population.

In cases of new HF, or where there is diagnostic uncertainty as to the cause of HF, CMR should be performed to evaluate the underlying aetiology.^21^ For example, it can detect myocardial ischaemia through stress perfusion imaging and quantify infiltration and infarction using late gadolinium enhancement analysis and parametric mapping. It is also the gold standard for the assessment of myocardial volume and mass, both of which are key metrics in patients with HF. Until recently, however, a major limitation of CMR has been the difficulty in evaluating diastolic function and estimating LVFP.

Central to this study was the use of a previously validated predictive model for estimating LVFP from easily acquired CMR metrics. This model has been subsequently upgraded to accommodate the influence of sex on model performance. The CMR‐modelled PCWP model is dependent on LAV and LVM, both established and highly reproducible independent predictors of PCWP, as determined by right‐heart catheterization.^13^, ^22^ The key advantage of this method of deriving an estimated PCWP is the ease of measuring LVM and LAV, with no requirement to obtain dedicated CMR sequences or to derive additional parameters from the images.

This study has demonstrated that hypertension, male sex, ageing (especially for those >70 years), obesity and regular consumption of alcohol are independently associated with a raised CMR‐modelled PCWP. These are expected findings, and further corroborate the robustness of the model used. The major effect of age on the vasculature is systemic hypertension, resulting from an increase in arterial stiffness and early wave reflections.^23^, ^24^, ^25^ This increase in afterload leads to ventricular‐arterial uncoupling and to a compensatory increase in LVM. This in turn leads to a loss of LV compliance and diastolic dysfunction, and to increased LAV, primarily due to LA remodelling.^22^ Even when accounting for common comorbid conditions (obesity, hypertension and diabetes), arterial stiffness is consistently greater in patients with HF than in those who, with similar comorbidities, do not have HF.^26^ Furthermore, evidence indicates that once the LV compliance drops significantly and the RV wall thickness increases, then disease may be no longer amenable to therapy.^27^ As such, a therapeutic opportunity may rest in identifying patients with preclinical diastolic dysfunction and who are at risk of HF. For instance, the SPRINT trial showed that the risk of incipient HF is reduced by 38% when SBP is targeted at <120 mmHg,^28^ compared with the standard target of <140 mmHg.^29^ Elevated BMI is also an established risk factor for new‐onset HF.^30^ It is a major determinant of arterial stiffness and is associated with concentric LV hypertrophy and LV dysfunction.^30^ To date, the relationship between obesity and clinical outcome has not explicitly been investigated within the obese HFpEF phenotype.^27^

This study sheds light on CMR‐modelled PCWP at a population level: by elucidating the risk factors for and prognostic significance of elevated CMR‐modelled PCWP, we can identify groups within the population at increased risk of raised filling pressures and therefore poor outcomes. In specific groups of patients with multiple risk factors for raised CMR‐modelled PCWP, there may be value in providing further tests to confirm the presence of elevated LVFP and thereby allow targeted intervention before the development of symptoms to reduce the incidence of adverse outcomes. Such a screening strategy, employing testing targeted to the high risk groups identified by this study, is particularly relevant for obese patients, in whom the sensitivity of NTproBNP is lowered, even in the context of markedly elevated LVFP.

To facilitate this approach, we have produced a nomogram mapping the absolute and relative risks of elevated LVFP according to sex, age, blood pressure and the presence or absence of obesity. For example, a 50 year‐old female with an SBP of 120 mmHg has a less than 5% risk of elevated CMR‐modelled PCWP, while a 80 year‐old obese male with an SBP of 160 mmHg has an absolute risk >30%, and a 12‐fold increased risk compared with the lowest risk males.

### Limitations

There is a healthy volunteer selection bias in the UKB cohort.^31^ Therefore, external validation in independent samples and other population cohorts should be conducted in future studies. The UKB imaging study consists predominantly of White British individuals, making generalizability to other ethnicities challenging, especially because there is a recognized difference in HF between different ethnic groups, often driven by variations in the prevalence of hypertension, diabetes mellitus and socioeconomic status.^32^ Furthermore, the relationship between CMR‐modelled and invasive LVFP has been explored and validated in only one study. The large sample size, alongside the fact we used CMR‐modelled PCWP as a dichotomous variable, adds robustness but does not reduce the risk of systematic bias. There is the potential for residual confounding that may have influenced the observed association between CMR‐modelled PCWP and clinical outcomes, despite adjusting for various covariates. We encourage future research to further investigate and clarify the complex relationships between variables, their underlying mechanisms, and the prognostic value of CMR‐modelling PCWP in predicting cardiovascular outcomes.

## Conclusions

CMR‐modelled PCWP is independently associated with incident HF and MACE. Independent risk factors for raised CMR‐modelled PCWP include age, male sex, hypertension, increased BMI and regular alcohol consumption. There is a rapid rise in CMR‐modelled PCWP after the age of 70 years. These findings may have implications for developing targeted screening strategies at the population level for HF.

## Funding

This work was supported by National Institute for Health Research (NIHR‐RP‐R3‐12‐027) and Wellcome Trust (220703/Z/20/Z and 215799/Z/19/Z). R. J. T. and C. G. C. are supported by NIHR Academic Clinical Fellowships.

N. A. recognizes the NIHR Integrated Academic Training programme, which supports his Academic Clinical Lectureship post and acknowledges the support from an Academy of Medical Sciences Starter Grant for Clinical Lecturers (Ref: SGL024\1024), which enabled the computational experiments. This research has been conducted using the UK Biobank Resource under Application 2964. The authors wish to thank all UK Biobank participants and staff. We acknowledge the British Heart Foundation for funding the manual analysis to create a cardiovascular magnetic resonance imaging reference standard for the UK Biobank imaging‐resource in 5000 CMR scans (PG/14/89/31194; S.E.P.). We also acknowledge support from the ‘SmartHeart’ Engineering and Physical Sciences Research Council programme grant (EP/P001009/1; S.E.P.). This work was part of the translational research portfolio of the National Institute for Health Research Biomedical Research Centre at Barts and The London School of Medicine and Dentistry; N. A. and S. E. P. acknowledge support from this centre.

## Conflict of interest statement

P. G. is a clinical advisor for Pie Medical Imaging and Medis Medical Imaging. S. E. P. is a consultant to Circle Cardiovascular Imaging. R. J. T. is an advisor to Panacea Innovation. All other authors have no conflicts of interest to declare.

## Supporting information


**Table S1.** ICD10 codes for heart failure.
**Table S2.** ICD10 codes for MACE.
**Table S3.** Non‐linear relationship between PCWP and age.
**Table S4.** Comparison of the performance of different models to predict outcomes.

## Data Availability

This research has been conducted using the UK Biobank resource. The raw data, derived data, analyses and results will be clearly annotated and returned to UK Biobank, which will then incorporate the returned data into the central repository. UK Biobank will make the data available to all bona fide researchers for all types of health‐related research that is in the public interest, without preferential or exclusive access for any person. All researchers will be subject to the same application process and approval criteria as specified by UK Biobank. Please see the UK Biobank website for detailed access procedure (www.ukbiobank.ac.uk/register‐apply/).
