# Supplementary material for: Risk factors for raised left ventricular filling pressure by cardiovascular magnetic resonance: Prognostic insights
Source: ESC Heart Fail. 2024 Aug 12;11(6):4148–59. doi: 10.1002/ehf2.15011 (PMC11631267; doi:10.1002/ehf2.15011)
Supplement: Supplementary file 1 — Table S1. ICD10 codes for heart failure. Table S2. ICD10 codes for MACE. Table S3. Non‐linear relationship between PCWP and age. Table S4. Comparison of the performance of different models to predict outcomes. [file EHF2-11-4148-s001.docx]

# Supplementary Document

**Table of contents**

**Contents**

[Supplementary Document 1](#_Toc165970754)

[Methods 2](#_Toc165970755)

[Cardiovascular magnetic resonance imaging 2](#_Toc165970756)

[CMR image analysis 2](#_Toc165970757)

[Supplementary Table 1. ICD10 codes for heart failure 3](#_Toc165970758)

[Supplementary Table 2. ICD10 codes for MACE 4](#_Toc165970759)

[Supplementary Table 3. Non-linear relationship between PCWP and age 8](#_Toc165970760)

[Supplementary Table 4. Comparison of the performance of different models to predict outcomes 10](#_Toc165970761)

## Methods

**CMR-modelled PCWP equation**

CMR PCWP = 5.7591 + (0.07505*LAV) + (0.05289*LVM) – (1.9927*sex) [female = 0; male = 1]

### Supplementary Table 1. ICD10 codes for heart failure

| I11 | Hypertensive heart disease with (congestive) heart failure |
| --- | --- |
| I13.0 | Hypertensive heart and renal disease with (congestive) heart failure |
| I13.2 | Hypertensive heart and renal disease with both (congestive) heart failure and renal failure |
| I25.5 | Ischaemic cardiomyopathy |
| I50.0 | Congestive heart failure |
| I50.1 | Left ventricular failure |
| I50.9 | Heart failure, unspecified |
| J81 | Pulmonary oedema |
| K76.1 | Chronic passive congestion of liver |

### Supplementary Table 2. ICD10 codes for MACE

| I21 | Acute myocardial infarction |
| --- | --- |
| I210 | I21.0 Acute transmural myocardial infarction of anterior wall |
| I211 | I21.1 Acute transmural myocardial infarction of inferior wall |
| I212 | I21.2 Acute transmural myocardial infarction of other sites |
| I213 | I21.3 Acute transmural myocardial infarction of unspecified site |
| I214 | I21.4 Acute subendocardial myocardial infarction |
| I219 | I21.9 Acute myocardial infarction, unspecified |
| I21X | I21.X Presumed acute myocardial infarction (unconfirmed) |
| I22 | I22 Subsequent myocardial infarction |
| I22.0 | Subsequent myocardial infarction of anterior wall |
| I22.1 | Subsequent myocardial infarction of inferior wall |
| I22.8 | Subsequent myocardial infarction of other sites |
| I22.9 | Subsequent myocardial infarction of unspecified site |
| I23 | Certain current complications following acute myocardial infarction |
| I23.0 | Haemopericardium as current complication following acute myocardial infarction |
| I23.1 | Atrial septal defect as current complication following acute myocardial infarction |
| I23.2 | Ventricular septal defect as current complication following acute myocardial infarction |
| I23.3 | Rupture of cardiac wall without haemopericardium as current complication following acute myocardial infarction |
| I23.4 | Rupture of chordae tendineae as current complication following acute myocardial infarction |
| I23.5 | Rupture of papillary muscle as current complication following acute myocardial infarction |
| I23.6 | Thrombosis of atrium, auricular appendage and ventricle as current complications following acute myocardial infarction |
| I23.8 | Other current complications following acute myocardial infarction |
| I24 | Other acute ischaemic heart diseases |
| I24.0 | Coronary thrombosis not resulting in myocardial infarction |
| I24.1 | Dressler's syndrome |
| I24.8 | Other forms of acute ischaemic heart disease |
| I24.9 | Acute ischaemic heart disease, unspecified |
| I25 | Chronic ischaemic heart disease |
| I25.0 | Atherosclerotic cardiovascular disease, so described |
| I25.1 | Atherosclerotic heart disease |
| I25.2 | Old myocardial infarction |
| I25.3 | Aneurysm of heart |
| I25.4 | Coronary artery aneurysm |
| I25.5 | Ischaemic cardiomyopathy |
| I25.6 | Silent myocardial ischaemia |
| I25.8 | Other forms of chronic ischaemic heart disease |
| I63.0 | Cerebral infarction due to thrombosis of precerebral arteries |
| I63.1 | Cerebral infarction due to embolism of precerebral arteries |
| I63.2 | Cerebral infarction due to unspecified occlusion or stenosis of precerebral arteries |
| I63.3 | Cerebral infarction due to thrombosis of cerebral arteries |
| I63.4 | Cerebral infarction due to embolism of cerebral arteries |
| I63.5 | Cerebral infarction due to unspecified occlusion or stenosis of cerebral arteries |
| I63.6 | Cerebral infarction due to cerebral venous thrombosis, nonpyrogenic |
| I63.8 | Other cerebral infarction |
| I63.9 | Cerebral infarction, unspecified |
| I64 | Stroke, not specified as haemorrhage or infarction |
| I65.0 | Occlusion and stenosis of vertebral artery |
| I65.1 | Occlusion and stenosis of basilar artery |
| I65.2 | Occlusion and stenosis of carotid artery |
| I65.3 | Occlusion and stenosis of multiple and bilateral precerebral arteries |
| I65.8 | Occlusion and stenosis of other precerebral artery |
| I65.9 | Occlusion and stenosis of unspecified precerebral artery |
| I66.0 | Occlusion and stenosis of middle cerebral artery |
| I66.1 | Occlusion and stenosis of anterior cerebral artery |
| I66.2 | Occlusion and stenosis of posterior cerebral artery |
| I66.3 | Occlusion and stenosis of cerebellar arteries |
| I66.4 | Occlusion and stenosis of multiple and bilateral cerebral arteries |
| I66.8 | Occlusion and stenosis of other cerebral artery |
| I66.9 | Occlusion and stenosis of unspecified cerebral artery |
| I69.3 | Sequelae of cerebral infarction |

### Supplementary Table 3. Non-linear relationship between PCWP and age

The dataset was randomly sampled to create training (75%) and test (25%) sets. Univariable ordinal least squares regression was performed using the training set, modelling PCWP against restricted cubic splines of age with 5, 7, 9, 11 and 13 knots. The performance of the models in the training and test sets were compared using mean absolute error, residual mean square error, R^2^ and Akaike information criterion. A model with 9 knots was chosen.

| Knots | Dataset | MAE | RMSE | R2 | AIC |
| --- | --- | --- | --- | --- | --- |
| 5 | Test set | 1.020 | 1.401 | 0.020 | 102,703 |
| 5 | Training set | 1.005 | 1.384 | 0.016 | 102,703 |
| 7 | Test set | 1.019 | 1.401 | 0.020 | 102,702 |
| 7 | Training set | 1.004 | 1.384 | 0.016 | 102,702 |
| 9 | Test set | 1.019 | 1.401 | 0.021 | 102,700 |
| 9 | Training set | 1.004 | 1.384 | 0.017 | 102,700 |
| 11 | Test set | 1.019 | 1.401 | 0.021 | 102,699 |
| 11 | Training set | 1.004 | 1.384 | 0.017 | 102,699 |
| 13 | Test set | 1.019 | 1.400 | 0.021 | 102,699 |
| 13 | Training set | 1.004 | 1.384 | 0.017 | 102,699 |

Multivariable ordinary least squares regression was performed using the training set, modelling PCWP against a restricted cubic spline of age with 9 knots, adjusting for sex, hypertension, hyperlipidaemia, diabetes, regular alcohol consumption and any smoking history. The modelled relationship between PCWP and age was inspected to identify the inflection point after which there was a steep increase in PCWP with incremental age. This modelling process was repeated in the validation set and a similar relationship and inflection point were observed.

### Supplementary Table 4. Comparison of the performance of different models to predict outcomes

| **Model** | **Degrees** | **AIC** | **Concordance** |
| --- | --- | --- | --- |
| **Incident heart failure** | | | |
| LVEF + PCWP | 6 | 3,819 | 0.821 |
| LVEF | 5 | 3,850 | 0.817 |
| LA volume + LV mass | 6 | 3,901 | 0.812 |
| PCWP | 5 | 3,929 | 0.796 |
| Clinical variables | 4 | 3,981 | 0.781 |
| **MACE** | | | |
| LA volume + LV mass | 6 | 13,848 | 0.728 |
| LVEF + PCWP | 6 | 13,882 | 0.718 |
| LVEF | 5 | 13,889 | 0.716 |
| PCWP | 5 | 13,893 | 0.717 |
| Clinical variables | 4 | 13,902 | 0.714 |
